# Mycobacterial Infections Mimicking Malignancy in Solitary Pulmonary Lesions: A Cone Beam CT‐Guided Biopsy Case Series

**DOI:** 10.1002/rcr2.70300

**Published:** 2025-08-03

**Authors:** Sammy Onyancha, Ramin Lonnes, Peter Hollaus, Waldemar Schreiner, Gernot Rohde

**Affiliations:** ^1^ Department of Pulmonology St. Elisabethen Krankenhaus Frankfurt Germany; ^2^ Department of Thoracic Surgery St. Elisabethen Krankenhaus Frankfurt Germany; ^3^ Goethe University Frankfurt, University Hospital Department of Thoracic Surgery Frankfurt Germany; ^4^ Department of Respiratory Medicine Universitätsklinikum Marburg Marburg Germany

**Keywords:** cone‐beam CT, granulomatous inflammation, mycobacterial infection, non‐tuberculous mycobacteria, solitary pulmonary nodule, tuberculoma

## Abstract

Solitary pulmonary lesions are often associated with malignancy. Cone beam computed tomography (CBCT) enhances bronchoscopic biopsy accuracy by confirming tool‐in‐lesion positioning. We present five cases of solitary pulmonary nodules initially suspected to be malignant based on imaging and clinical context. Despite clear tool‐in‐lesion confirmation via CBCT, initial pathology was non‐diagnostic for malignancy. Upon further microbiological analysis, four cases were diagnosed as mycobacterial infections. A fifth case, which underwent surgical resection due to persistent diagnostic uncertainty, was subsequently found to harbour mycobacterial infection; retrospective review of the original biopsy also confirmed this. These cases highlight the importance of including mycobacterial infections such as tuberculoma in the differential diagnosis of solitary pulmonary nodules and stress the need for comprehensive microbiological evaluation in CBCT‐confirmed biopsies, especially when histology is non‐malignant. Our findings also emphasise the potential diagnostic utility of microbiological tests—including PCR—even prior to histology review when CBCT confirms tool‐in‐lesion. This approach may prevent unnecessary surgical interventions and associated morbidity.

## Graphical abstract

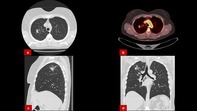

## 1 Introduction

Solitary pulmonary lesions are often evaluated with high suspicion for malignancy, particularly in high‐risk populations [1]. Cone beam computed tomography (CBCT) has emerged as a powerful adjunct to navigational bronchoscopy, enhancing diagnostic accuracy by confirming biopsy tool placement within the target lesion [2]. While non‐diagnostic or benign pathology results may raise concerns about sampling error, CBCT confirmation should prompt consideration of alternative diagnoses rather than immediate escalation to invasive procedures. Mycobacterial infections, though uncommon in high‐income settings, can radiographically and clinically mimic malignancy [3, 4, 5]. This case series illustrates how mycobacterial infections presented as solitary nodules and were accurately biopsied using CBCT‐guided techniques, underscoring the importance of thorough microbiological evaluation in such contexts.

## 2 Case Series

Five patients with peripheral pulmonary lesions underwent CBCT‐guided navigational bronchoscopy at our institution. CBCT‐guided cryobiopsy was selected in these cases due to the peripheral location and small size of the lesions, which were considered challenging for conventional transbronchial forceps biopsy. Additionally, the risk of pneumothorax from CT‐guided percutaneous biopsy, especially in upper lobe lesions, made bronchoscopic cryobiopsy a safer alternative. The ability of CBCT to confirm tool‐in‐lesion placement in real time further justified its use for these cases.

### 2.1 Case 1

**FIGURE 1:**
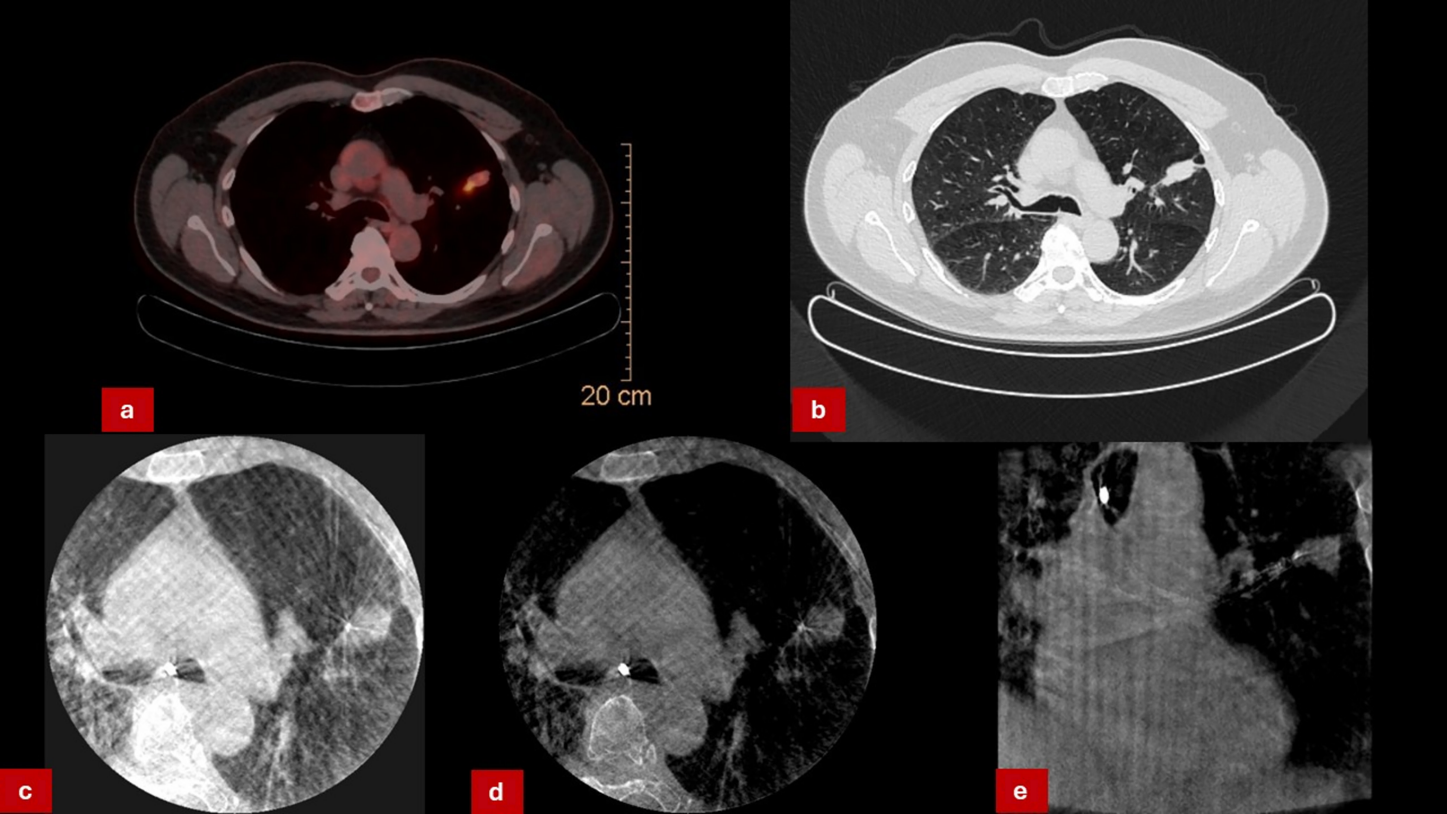
(a) PET‐CT scan of suspicious left upper lobe lesion. (b) CT‐scan showing suspicion of lung cancer in the left lung. (c–e) CBCT‐Imaging showing “Tool‐in‐lesion” (1.1 mm cryoprobe placed within suspicious lesion).

A 57‐year‐old male, ex‐smoker with a known history of HIV infection presented with a 1.8 cm left upper lobe nodule discovered on routine imaging. PET‐CT showed moderate uptake (SUV 4.5). CBCT‐guided bronchoscopy confirmed tool‐in‐lesion, and biopsies were obtained (Figure 1). Histopathology revealed granulomatous inflammation without evidence of malignancy. Initial interpretation deemed the biopsy non‐conclusive. Given the patient's history of HIV infection, PCR for *Mycobacterium tuberculosis* and non‐tuberculous mycobacteria was performed following histological suspicion of granulomatous disease, seeing as no PCR was used at initial sampling. Cultures subsequently grew *Mycobacterium kansasii* , prompting initiation of an antimycobacterial treatment. Final diagnosis via culture was available 21 days after bronchoscopy.

### 2.2 Case 2

**FIGURE 2:**
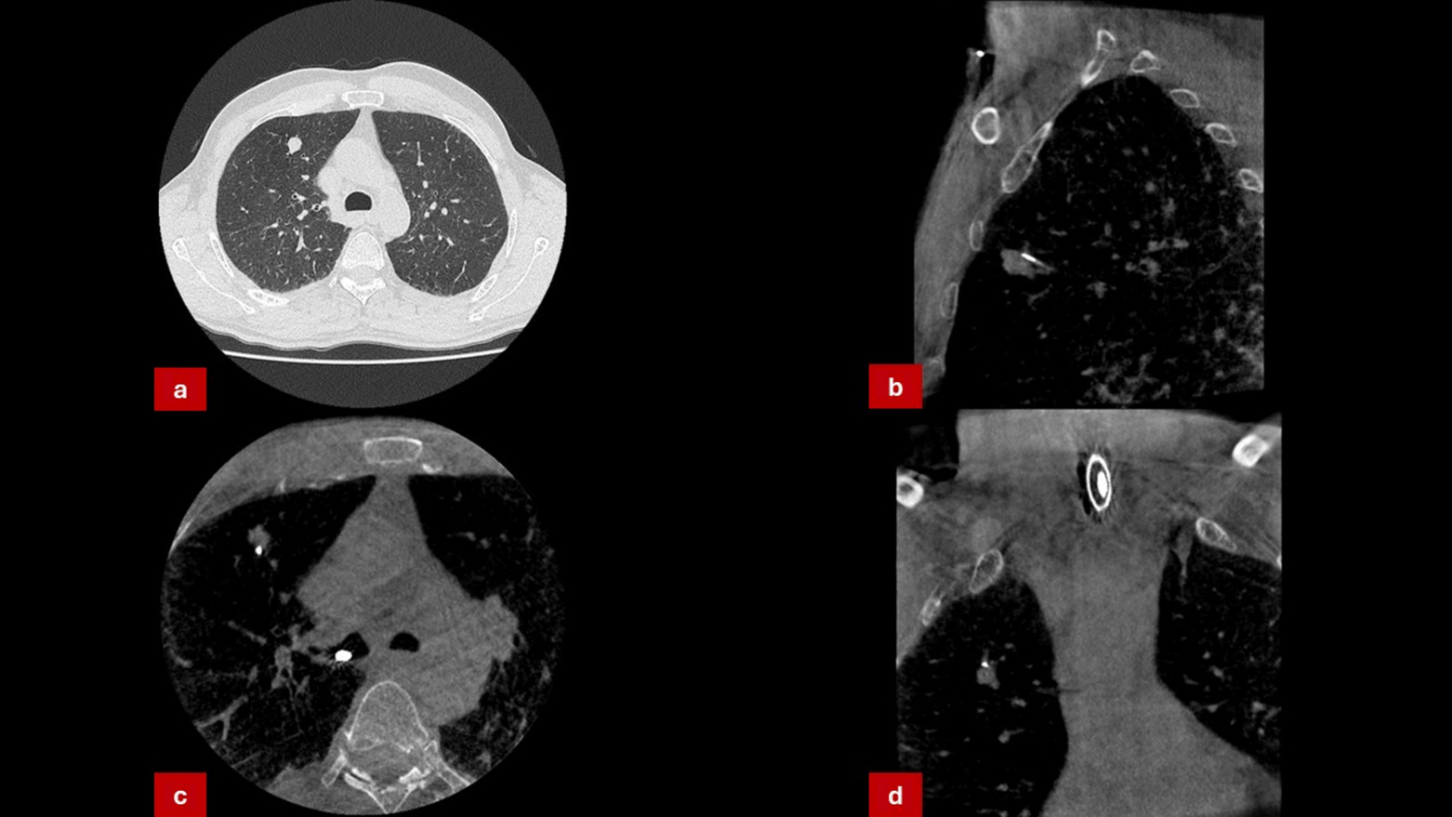
(a) CT‐scan showing solitary lesion in the right upper lobe. (b–d) CBCT‐Imaging confirming precise placement of biopsy tool (1.1 mm cryoprobe) within the targeted lesion.

A 63‐year‐old man, active smoker, with no prior TB history presented with a 1.4 cm peripheral right upper lobe nodule. Given his risk profile, malignancy was suspected. Navigational bronchoscopy was performed and CBCT‐guided biopsy again confirmed tool‐in‐lesion (Figure 2). Histology showed necrotising granulomatous inflammation without malignancy. Ziehl‐Neelsen staining and cultures returned positive for *Mycobacterium avium* complex. He was managed with antimycobacterial therapy without the need for surgical intervention. Final microbiological diagnosis was established 28 days post‐biopsy.

### 2.3 Case 3

**FIGURE 3:**
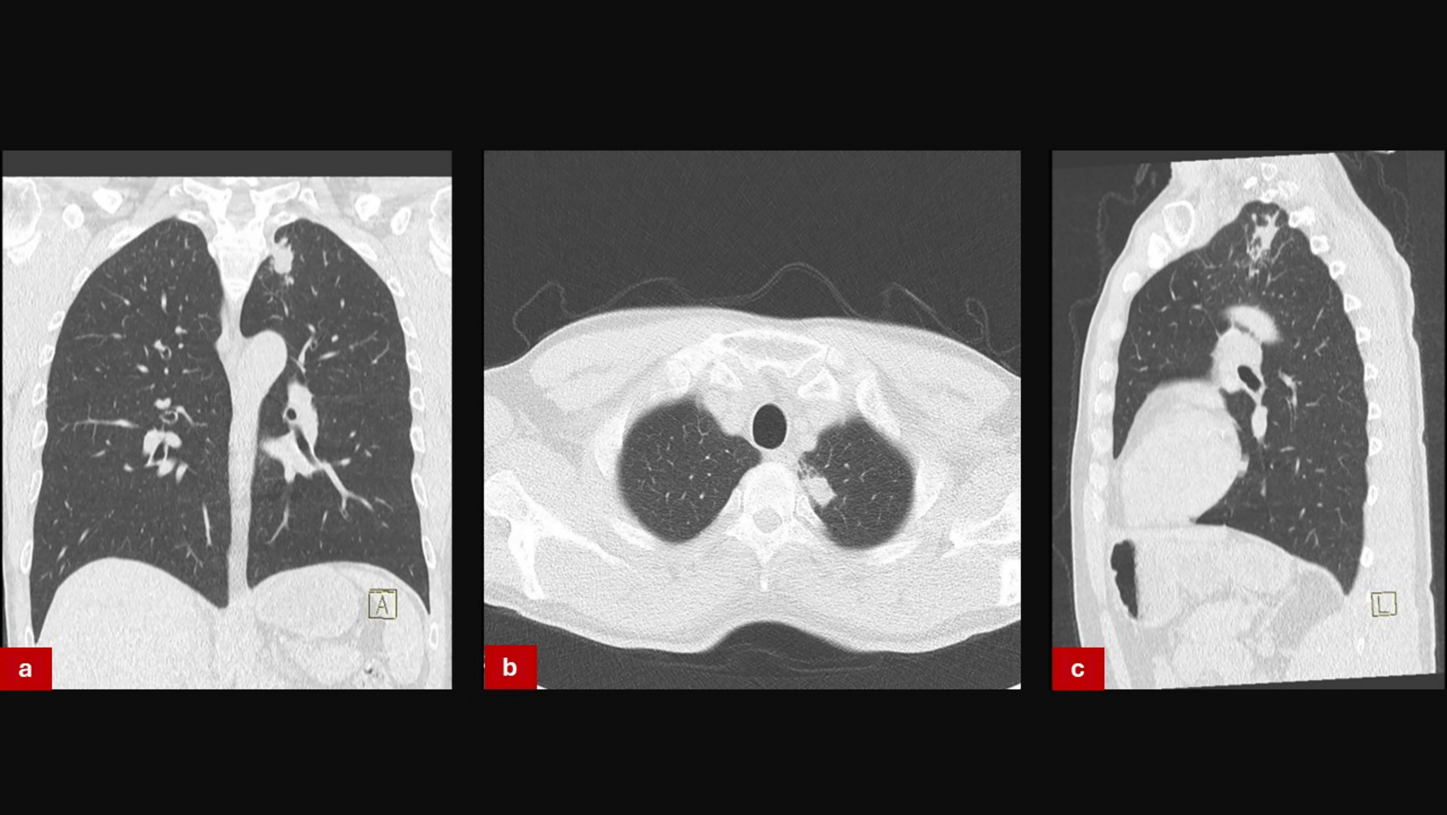
(a–c) CT‐scan of solitary mass in left upper lobe.

A 69‐year‐old man with a history of COPD presented with a 1.2 cm left upper lobe lesion (Figure 3). CBCT‐assisted bronchoscopy showed precise sampling of the lesion. Biopsies showed caseating granulomas. No malignant cells were found. In this case, PCR for *Mycobacterium tuberculosis* was conducted concurrently with histological analysis due to suggestive imaging and given the prior experience of positive findings in solitary pulmonary lesions. PCR for *Mycobacterium tuberculosis* complex returned positive. Final diagnosis was available within 7 days. Treatment was initiated with standard four‐drug antitubercular therapy.

### 2.4 Case 4

**FIGURE 4:**
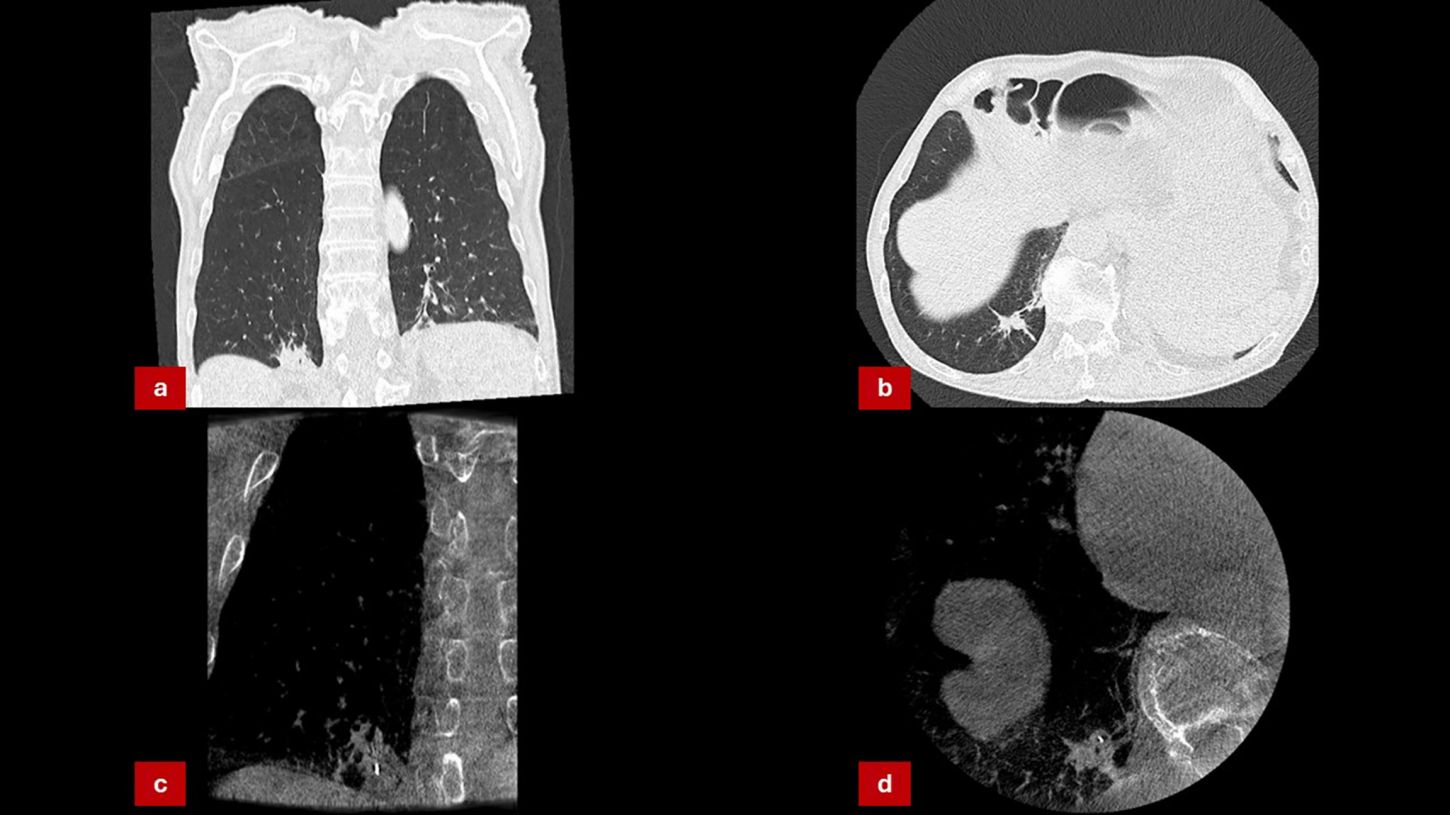
(a, b) CT scans revealing a solitary pulmonary lesion within the left lower lobe. (c, d) CBCT‐Imaging done during bronchoscopy confirming appropriate tool placement.

A 67‐year‐old male, active smoker, presented with a 1.3 cm right lower lobe lesion. Given his clinical symptoms—including significant weight loss and night sweats—malignancy was suspected. Bronchoscopy with CBCT‐assisted navigation confirmed accurate tool placement within the centre of the lesion. Histopathology revealed focal necrotising inflammation without any signs of malignancy. Routine PCR testing was conducted following pathology showing necrotising inflammation, and the microbiological analysis identified *Mycobacterium tuberculosis* , confirming a diagnosis of tuberculoma 6 days after the initial biopsy. The patient was started on antitubercular therapy with clinical and radiological improvement (Figure 4).

### 2.5 Case 5

**FIGURE 5:**
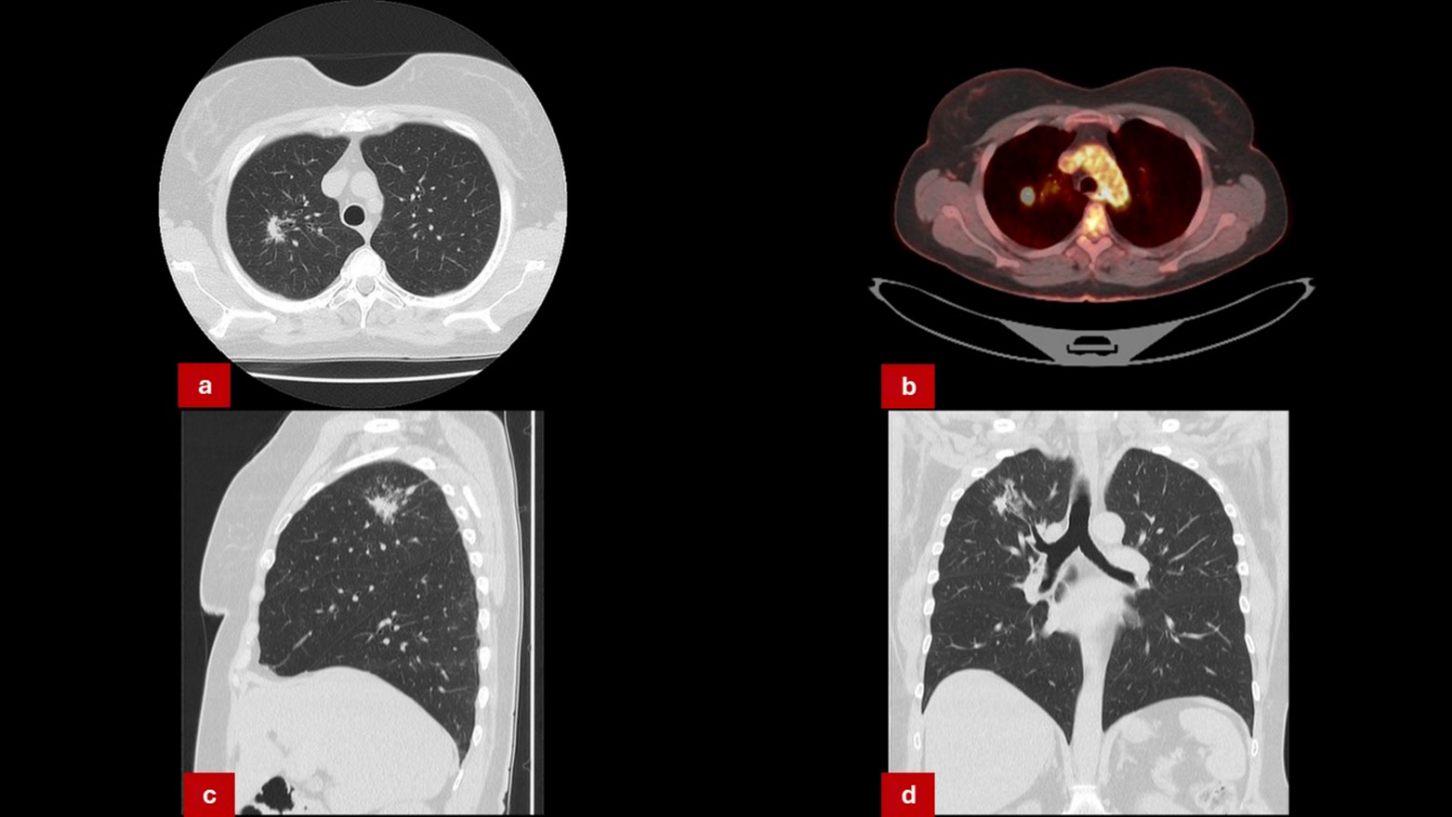
(a, c, d) CT‐scan revealing suspicious lesion in right upper lobe. (b) PET‐CT scan showing avid uptake in the right upper lobe lesion.

A 55‐year‐old woman with a solitary 1.3 cm right upper lobe nodule underwent CBCT‐guided bronchoscopy. Tool‐in‐lesion was confirmed, but histopathology was interpreted as non‐diagnostic, revealing necrotic debris and chronic inflammation. Repeat bronchoscopy was considered; however, due to persistent clinical suspicion for malignancy owing to high PET avidity (Figure 5) and ongoing patient anxiety, video‐assisted thoracoscopic surgical (VATS) resection was performed. Resected tissue confirmed a diagnosis of *Mycobacterium tuberculosis* infection 9 days after operation. Upon retrospective review, the original bronchoscopic biopsy was found to have the same organism on extended microbiologic testing.

## 3 Discussion

These five cases illustrate that mycobacterial infections—including *Mycobacterium tuberculosis* and non‐tuberculous mycobacteria (NTM)—can closely mimic malignancy in solitary pulmonary nodules. Despite strong pre‐test probability of malignancy, accurate CBCT‐guided sampling revealed benign granulomatous disease. The initial impression of “non‐conclusive” biopsies was revised upon microbiological analysis, highlighting the importance of integrating CBCT findings into the diagnostic workflow. When tool‐in‐lesion is confirmed, clinicians should be cautious in interpreting “non‐diagnostic” pathology as sampling failure.

Our experience shows that CBCT‐guided cryobiopsy is feasible even in lesions as small as 1.2 cm when precise tool‐in‐lesion positioning is confirmed. Lesions smaller than 1 cm were not included in our series, but existing literature suggests that real‐time imaging guidance significantly enhances diagnostic success even at such small sizes. The average procedure time in our series was approximately 50–65 min, with only a minor complication of self‐resolving pneumothorax occurring in case 1 being recorded. Major complications such as significant bleeding or large pneumothorax requiring intervention were not observed in this series.

At our centre, PCR testing for *Mycobacterium tuberculosis* and non‐tuberculous mycobacteria (NTM) is routinely performed on lymph node biopsies as well as in the microbiological analysis of bronchial secretions. However, it is not consistently applied to transbronchial lung biopsies during the initial diagnostic workup. In the case series, PCR testing was typically introduced only after histological findings indicated granulomatous inflammation. Implementing a more standardised diagnostic approach that includes early PCR testing may help expedite diagnosis and reduce diagnostic uncertainty.

The time from initial biopsy to final microbiological diagnosis ranged from 7 to 28 days. This delay can challenge clinical decision‐making, particularly when PET avidity or patient anxiety pressures towards expedited intervention.

While no single radiologic feature definitively distinguishes mycobacterial infection from malignancy, the presence of well‐defined nodules with central cavitation or tree‐in‐bud patterns may raise suspicion, especially in the absence of lymphadenopathy or metastasis.

The role of ROSE, though underutilised in our series, may be critical. In retrospect, it could have provided earlier clues toward granulomatous inflammation, particularly in Case 5, potentially avoiding surgical escalation.

These cases emphasise three key points:

1. Mycobacterial infections should be considered in the differential diagnosis of solitary pulmonary nodules, even in regions of low prevalence.
2. CBCT‐guided bronchoscopy provides high‐confidence biopsy placement, which should increase trust in the tissue result, prompting deeper investigation into infectious aetiologies when malignancy is not found.
3. Premature escalation to surgical resection may be avoidable if microbiological studies are thoroughly pursued following a CBCT‐confirmed non‐malignant biopsy.

Given the diagnostic yield in our cases, we now recommend routine submission of bronchoscopic samples for mycobacterial culture and PCR testing when CBCT confirms tool‐in‐lesion and histology is non‐malignant. This step is particularly important in patients without classic risk factors or radiological signs of infection. Such an approach may accelerate diagnosis, avoid unnecessary surgical procedures, and guide timely treatment initiation.

## Author Contributions

All the authors contributed to the manuscript. The first draft of the manuscript was written by Sammy Onyancha, and all the authors commented on previous versions of the manuscript. All the authors have read and approved the final manuscript.

## Consent

Written informed consent was obtained from the patients for publication of this case series and any accompanying images.

## Conflicts of Interest

The authors declare no conflicts of interest.

## Data Availability

The data that support the findings of this study are available from the corresponding author upon reasonable request.
